# Adipose tissue-derived extracellular vesicles from obese mice suppressed splenocyte-mediated pancreatic cancer cell death

**DOI:** 10.29219/fnr.v68.10545

**Published:** 2024-10-03

**Authors:** Inae Jeong, Shinjung Park, Jinbum Park, Ok-Kyung Kim

**Affiliations:** Division of Food and Nutrition, Chonnam National University, Gwangju, Republic of Korea

**Keywords:** obesity, adipose tissue, extracellular vesicles, pancreatic cancer, immunity

## Abstract

**Background:**

Obesity is a risk factor for pancreatic cancer and negatively contributes to the immune system. However, the mechanisms by which obesity mediates these actions are still poorly understood. Recent studies have demonstrated that extracellular vesicles (EVs) are key mediators of communication between cells and may influence various aspects of cancer progression.

**Objectives:**

We aim to explore the influence of EVs derived from adipose tissue of obese mice on cytokine production within the interactions between cancer cells and immune cells.

**Design:**

We isolated EVs from the adipose tissue of both C57BL6/J mice and *Ob/Ob* mice. Subsequently, we treated EVs with Panc02 cells, the murine ductal pancreatic cancer cell line, which were co-cultured with splenocytes. Viability and SMAD4 gene expression were examined in Panc02 cells, and cytokine concentrations of IL-6, IL-4, IL-12, and IL-12p70 were measured in the cultured medium.

**Results:**

Interestingly, we observed a significant reduction in splenocyte-mediated Panc02 cell death when treated with EVs derived from the adipose tissue of *Ob/Ob* mice, compared to those from C57BL6/J mice. Additionally, EVs from *Ob/Ob* mice-derived adipose tissue significantly increased the levels of IL-4, IL-2, and IL-12p70 in the culture media of Panc02 cells co-cultured with splenocytes, compared to EVs from C57BL6/J mice-derived adipose tissue.

**Conclusion:**

Adipose tissue-derived EVs from obese mice suppressed splenocyte-mediated Panc02 cell death and upregulated IL-4, IL-2, and IL-12p70 in cultured medium.

## Popular scientific summary

- Adipose tissue-derived extracellular vesicles (EVs) from obese mice suppressed splenocyte-mediated Panc02 cell death and SMAD4 expression.
- The level of IL-4, IL-2, and IL-12p70 were upregulated in the culture media from Panc02 cells co-cultured with splenocytes.
- This is the first report exploring the role of adipose tissue-derived EVs in understanding the complex relationship between obesity, cancer, and immune responses.

Obesity, characterised by an excessive accumulation of adipose tissue, has reached alarming proportions worldwide (1). It is now considered one of the most prevalent and challenging public health issues of our time. The implications of obesity extend far beyond its established associations with metabolic disorders such as type 2 diabetes and cardiovascular disease (2, 3). Emerging evidence has illuminated a concerning link between obesity and an increased risk of various cancers, with pancreatic cancer being a prominent concern (4–6).

Pancreatic cancer, a highly aggressive and notoriously lethal malignancy, represents a significant global health burden. Its incidence and mortality rates continue to rise, underscoring the urgency of understanding its etiological factors (7, 8). Recent research endeavours have been dedicated to unravelling the intricate relationship between obesity and pancreatic cancer. While substantial progress has been made in elucidating the link between obesity and pancreatic cancer, these studies are focussed on aggressiveness of pancreatic cancer mediated by various pro-inflammatory cytokines and adipokines from adipose tissue, and there remain intricate facets of this association that require further investigation (9–14).

In recent years, studies have illuminated the multifaceted role of EVs in tumour growth, underscoring their influence on various aspects of cancer progression (15–18). These small, lipid bilayer-enclosed structures, released by cells into their surroundings, hold pivotal functions in intercellular communication and are implicated in a spectrum of physiological and pathological processes (17). Remarkably, despite their recognised significance, no study has yet unveiled the specific contribution of EVs to the interplay between obesity and pancreatic cancer.

Moreover, contemporary research on the interactions between cancer and the immune system has revealed how obesity can detrimentally affect the immune response to cancer through several pathways (19, 20). In light of this, our study aims to validate the impact of adipose tissue-derived EVs from obese mice on the aggressiveness of pancreatic cancer. Additionally, we aim to explore the influence of these EVs on cytokine production within the interactions between cancer cells and immune cells. Through these investigations, we endeavour to shed light on the intricate mechanisms underlying the effect of adipose tissue-derived EVs on the pancreatic cancer-immune microenvironment in the context of obesity.

## Materials and methods

### Animals

Six-week-old male C57BL/6J mice and 6-week-old male *Ob/Ob* mice were obtained from Orientbio (Seongnam, Korea). They were fed a normal conventional chow diet for 6 weeks. All the animals were housed in a controlled environment at 22–25°C and kept under a 12 h light/dark cycle with free access to drinking water and chow. The animal study protocol was approved by the Institutional Animal Care and Use Committee of Chonnam National University (CNU IACUC-YB-R-2021-141), and the animals were maintained in accordance with the ‘Guidelines for Animal Experiments’ established by the university.

### Isolation of adipose tissue-derived EVs from mice

Subcutaneous, epididymal and visceral white adipose tissues from mice were collected, cut into small pieces, and cultured in Dulbecco’s modified Eagle medium (DMEM) containing 10% FBS for 48 h. The supernatant was collected and sequentially filtered through filters with pore sizes of 0.45 and 0.22 μm. And then, pure EVs were isolated using ExoQuick-TC^TM^ precipitation reagent (System biosciences) according to the manufacturer’s instructions. Isolated EVs were finally evaluated by nanoparticle tracking analysis (NTA) using NanoSight LM10 system (Malvern Panalytical Ltd, Malvern, UK) and CD63 protein expression.

### Cell culture

Panc02 cells were maintained in DMEM (Gibco) containing 10% foetal bovine serum (FBS; Gibco) and 1% penicillin/streptomycin (Gibco).

Splenocytes were isolated from mice spleen. Briefly, the spleen was ground using a 40 μm cell strainer (Corning). The precipitated cells were treated with red blood cell lysis buffer (Sigma). Splenocytes were then washed with phosphate-buffered saline (PBS) and resuspended in Roswell Park Memorial Institute medium (RPMI-1640, Gibco) containing 10% FBS and 1% penicillin/streptomycin. All cells were always incubated at 37°C under a humidified atmosphere consisting of 95% air and 5% carbon dioxide.

In the co-culture assay, splenocytes were seeded to Panc02 cells at 500 times the number of Panc02 cells inoculated. 50 μg/mL of EVs were administered at 0 and 24 h, and the assay was performed after 48 h.

### 4’6-diamidino-2-phenylindole (DAPI) staining and cell viability assay

Morphological observation of cell death was performed using DAPI staining. Cells were fixed with 4% paraformaldehyde (Sigma) for 10 min and permeabilised with 0.3% Triton X-100 (Sigma) for 1 h at 4°C. Subsequently, cell nuclei were labelled with ProLong^TM^ Gold Antifade Mountant with DAPI (Invitrogen).

Quantitative measure of cell death was conducted using the water-soluble tetrazolium salt-based EZ-Cytox assay kit (Dogenbio) according to the manufacturer’s instructions. In this assay, splenocytes were removed and only Panc02 cells were measured.

### Enzyme-linked immunosorbent assay

The medium in which Panc02 cells and splenocytes were co-cultured was harvested. After removing cell debris, the medium was used for enzyme-linked immunosorbent assay (ELISA). The cytokines IL-2, IL-4, IL-6 and IL-12p70 were measured using the DuoSet ELISA kit (R&D systems) according to the manufacturer’s instructions.

### RNA isolation and quantitative real-time PCR

RNA from Panc02 cells was extracted using RNeasy mini kit (Qiagen) according to the manufacturer’s instructions. 100 ng of RNA was used to synthesise the complementary DNA (cDNA) and iScript cDNA synthesis kit (Bio-rad) was used. Each sample was amplified using a iQ SYBR Green Supermix (Bio-rad), and suppressor of mothers against decapentaplegic 4 (*SMAD4*) primers (Forward: *5’-CAGGACAGCAGCAG AATGGA-3’*, Reverse: *5’-CGTTTTGGTGGTGAGGCA AA-3’*, Accession number: *NM_0085 40.3*) was used. cDNA was amplified using the CFX Duet Real-Time PCR System (Bio-Rad, Hercules, CA, USA).

### Statistical analysis

Results are presented as the mean ± standard deviation (SD). Data analysis was conducted using a Student’s *t*-test to compare two groups. Statistical significance was defined as *P* < 0.05.

## Results

### Effect of adipose tissue-derived EVs on splenocytes-mediated Panc02 cells death

**Fig. 1:**
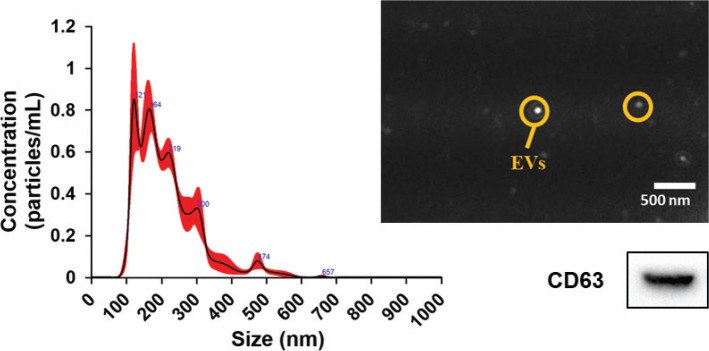
Size and vesicle observation using Nanosight and CD63 (cluster of differentiation) expression of extracellular vesicles from the adipose tissue.

Extracellular vesicles were isolated from the adipose tissues, and the vesicle size and the expression of the EVs marker CD63 was determined. We found a high peak value between 100–200 nm, corresponding to the size of conventional EVs and the expression of CD63 was confirmed in EVs isolated from adipose tissue (Fig. 1).

**Fig. 2:**
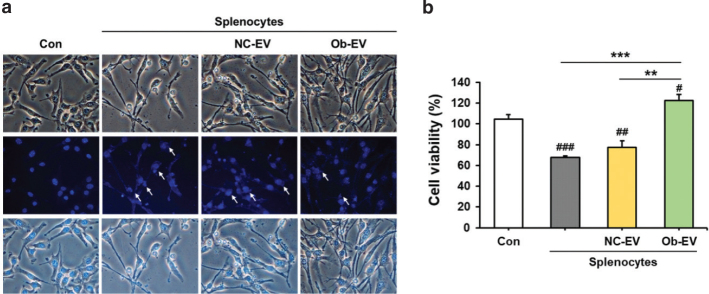
Effect of adipose tissue-derived extracellular vesicles (EVs) on the viability of Panc02 cells co-cultured with splenocytes. Co-cultures of splenocytes and Panc02 cells were treated NC-EV (adipose tissue derived extracellular vesicles from C57BL/6J mice) or Ob-EV (adipose tissue derived extracellular vesicles from *Ob/Ob* mice) and confirmed the fluorescence microscope images for the nuclei stained with DAPI (4’,6-diamidino-2-phenylindole) (blue) (a) and cell viability (b). Values are presented as the mean ± SD (*n* = 3). Con versus groups; *#P* < 0.05, *##P* < 0.01, *###P* < 0.001. **P < 0.01, ***P < 0.001.

We incubated EVs derived from the adipose tissue of C57BL6/J mice and *Ob/Ob* mice with Panc02 cell co-cultured splenocytes, and subsequently assessed the viability of Panc02 cells. The presence of co-cultured splenocytes in the Panc02 cell culture resulted in a suppression of cell viability when compared to cultures consisting solely of Panc02 cells (*P* < 0.001). Interestingly, when treated with EVs derived from the adipose tissue of *Ob/Ob* mice, splenocyte-mediated cell death in Panc02 cells was significantly reduced compared to treatment with EVs from the adipose tissue of C57BL6/J mice (*P* < 0.01) (Fig. 2). This finding indicates that EVs derived from adipose tissue during obesity development can inhibit the immune response against pancreatic cancer.

### Effect of adipose tissue-derived EVs on SMAD4 expression in Panc02 cells co-cultured with splenocytes

**Fig. 3:**
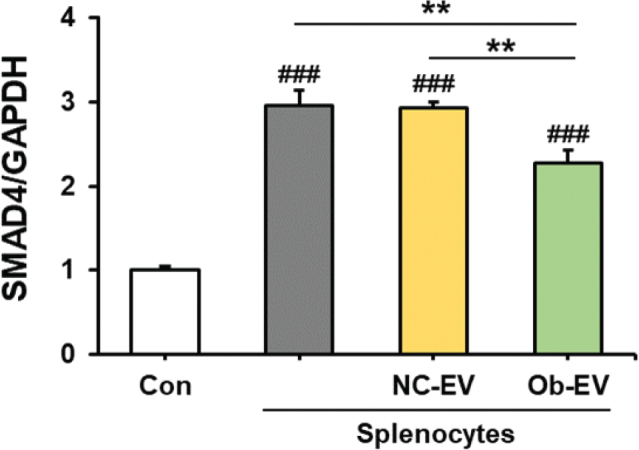
Effect of adipose tissue-derived extracellular vesicles (EVs) on mothers against decapentaplegic homolog 4 (*SMAD4*) expression in Panc02 cells co-cultured with splenocytes. Co-cultures of splenocytes and Panc02 cells were treated with NC-EV (adipose tissue derived extracellular vesicles from C57BL/6J mice) or Ob-EV (adipose tissue derived extracellular vesicles from *Ob/Ob* mice) and confirmed the *SMAD4* expression in Panc02 cells. Values are presented as the mean ± SD (*n* = 3). Con versus groups; *###P* < 0.001. **P < 0.01.

We investigated the effect of adipose tissue-derived EVs from C57BL6/J mice and *Ob/Ob* mice on *SMAD4* expression, identified as a tumour suppressor gene, in Panc02 cells co-cultured with splenocytes. The presence of co-cultured splenocytes in the Panc02 cell culture significantly increased *SMAD4* expression compared to cultures consisting solely of Panc02 cells (*P* < 0.001). Adipose tissue-derived EVs from *Ob/Ob* mice significantly decreased SMAD4 expression compared to adipose tissue-derived EVs from C57BL6/J mice (*P* < 0.01) (Fig. 3).

### Effect of adipose tissue-derived EVs on the levels of IL-6 in culture media from Panc02 cells co-cultured with splenocytes

**Fig. 4:**
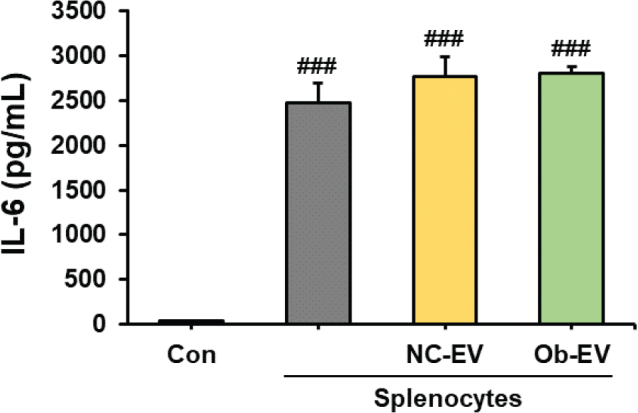
Effect of adipose tissue-derived extracellular vesicles (EVs) on IL-6 concentration in culture media from Panc02 cells co-cultured with splenocytes. Co-cultures of splenocytes and Panc02 cells were treated with NC-EV (adipose tissue derived extracellular vesicles from C57BL/6J mice) or Ob-EV (adipose tissue derived extracellular vesicles from *Ob/Ob* mice) and confirmed the level of IL-6. Values are presented as the mean ± SD (*n* = 3). Con versus groups; *###P* < 0.001.

We investigated the effect of adipose tissue-derived EVs from C57BL6/J mice and *Ob/Ob* mice on IL-6, cytokine-associated tumour-promoting inflammation, in culture media from Panc02 cells co-cultured with splenocytes. The presence of co-cultured splenocytes in the Panc02 cell culture significantly increased the level of IL-6 compared to cultures consisting solely of Panc02 cells (*P* < 0.001). When comparing the effects of adipose tissue-derived EVs from C57BL6/J mice and *Ob/Ob* mice on the level of IL-6, we found no significant difference (Fig. 4).

### Effect of adipose tissue-derived EVs on the levels of IL-4 in culture media from Panc02 cells co-cultured with splenocytes

**Fig. 5:**
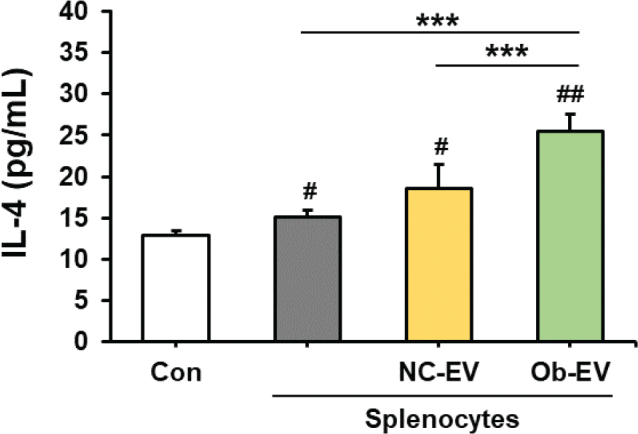
Effect of adipose tissue-derived extracellular vesicles (EVs) on interleukin 4 (IL-4) concentration in culture media from Panc02 cells co-cultured with splenocytes. Co-cultures of splenocytes and Panc02 cells were treated with NC-EV (adipose tissue derived extracellular vesicles from C57BL/6J mice) or Ob-EV (adipose tissue derived extracellular vesicles from *Ob/Ob* mice) and confirmed the level of IL-4. Values are presented as the mean ± SD (*n* = 3). Con versus groups; *#P* < 0.05, *##P* < 0.01. ***P < 0.001.

We investigated the effect of adipose tissue-derived EVs from C57BL6/J mice and *Ob/Ob* mice on IL-4, which plays a role in the tumour microenvironment and exhibits immune-suppressive properties, in culture media from Panc02 cells co-cultured with splenocytes. The presence of co-cultured splenocytes in the Panc02 cell culture significantly increased the level of IL-4 compared to cultures consisting solely of Panc02 cells (*P* < 0.05). Surprisingly, we found that adipose tissue-derived EVs from *Ob/Ob* mice significantly increased the level of IL-4 compared to adipose tissue-derived EVs from C57BL6/J mice (*P* < 0.001) (Fig. 5).

### Effect of adipose tissue-derived EVs on the levels of IL-2 and IL-12 in culture media from Panc02 cells co-cultured with splenocytes

**Fig. 6:**
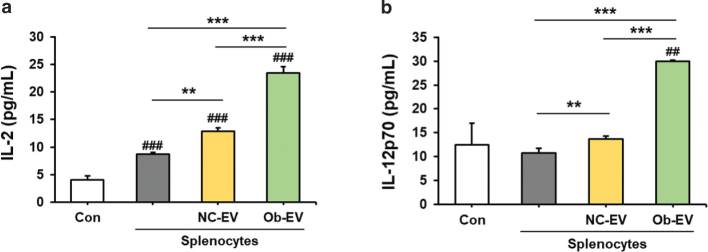
Effect of adipose tissue-derived extracellular vesicles (EVs) on IL-2 (interleukin 2) and IL-12 concentration in culture media from Panc02 cells co-cultured with splenocytes. Co-cultures of splenocytes and Panc02 cells were treated with NC-EV (adipose tissue derived extracellular vesicles from C57BL/6J mice) or Ob-EV (adipose tissue derived extracellular vesicles from *Ob/Ob* mice) and confirmed the level of IL-2 and IL-12. Values are presented as the mean ± SD (*n* = 3). Con versus groups; *##P* < 0.01, *###P* < 0.001. **P < 0.01, ***P < 0.001.

We measured the levels of IL-2 and IL-12 in the culture media from Panc02 cells co-cultured with splenocytes treated with adipose tissue-derived EVs from C57BL6/J mice or *Ob/Ob* mice. IL-2 and IL-12 activate an effector immune response against tumour cells, making them well-known potent cytokines for cancer immunotherapy. Adipose tissue-derived EVs from C57BL6/J mice significantly increased the levels of IL-2 and IL-12p70 in culture media from Panc02 cells co-cultured with splenocytes compared to the non-treatment group (*P* < 0.01). We expected that adipose tissue-derived EVs from *Ob/Ob* mice would result in reduced levels of IL-2 and IL-12p70 in the culture media from Panc02 cells co-cultured with splenocytes. However, interestingly, adipose tissue-derived EVs from *Ob/Ob* mice significantly increased the levels of IL-2 and IL-12p70 compared to adipose tissue-derived EVs from C57BL6/J mice (*P* < 0.001) (Fig. 6).

## Discussion

Cytokines, signalling molecules secreted by various immune cells, orchestrate the intricate dance of the immune system’s response to cancer. They serve as messengers, transmitting vital information that influences immune cell behaviour and activity. Certain cytokines act as key mediators in shaping the tumour microenvironment and have a multifaceted role in cancer immunosurveillance which is a critical mechanism by which the immune system actively seeks out and destroys emerging cancer cells within the body. They can either promote or inhibit immune responses, and their balance is critical in determining the fate of cancer cells (21–23). Cytokines regulate the proliferation, differentiation, and activation of immune cells, including cytotoxic T lymphocytes (CTLs), natural killer (NK) cells, and macrophages, all of which are instrumental in recognising and eradicating cancerous cells (24–26). Understanding the dynamic interplay between cytokines and the immune system in the context of shaping the tumour microenvironment and cancer immunosurveillance is essential for unravelling the complexities of tumour development and for devising targeted immunotherapies. In this study, we aim to investigate the changes in cytokines in the immune response to pancreatic cancer, particularly within the context of obesity-induced alterations in the immune microenvironment. Firstly, we observed a significant reduction in splenocyte-mediated Panc02 cell death, and we also confirmed reduced expression of *SMAD4*, a tumour suppressor gene, when treated with EVs derived from the adipose tissue of *Ob/Ob* mice. Therefore, we proceeded to measure the cytokines in the culture media from Panc02 cells co-cultured with splenocytes.

In some cancers, elevated levels of IL-6 have been associated with tumour progression, increased invasiveness and resistance to certain therapies. IL-6 can promote cell survival, proliferation and migration, all of which are key processes in cancer progression. Additionally, IL-6 can contribute to chronic inflammation, which is a hallmark of cancer development (27, 28). Bent et al. suggested that IL-6 inhibition can switch cancer cell clearance from primarily apoptotic to immunogenic, promoting and maintaining durable anti-tumour immune responses (29). Thus, we investigated the level of IL-6 in the culture media from Panc02 cells co-cultured with splenocytes. However, EVs derived from the adipose tissue of obese mice did not affect the level of IL-6, indicating that the function of adipose tissue-derived EVs in splenocyte-mediated Panc02 cell death is not dependent on IL-6.

Interestingly, EVs derived from the adipose tissue of obese mice led to an increase in the level of IL-4 in the culture media from Panc02 cells co-cultured with splenocytes. Elevated levels of IL-4 are commonly observed in cancer patients, and the up-regulation of IL-4/IL-4R signalling has been demonstrated to promote cancer cell proliferation, apoptotic resistance and metastatic potential within the tumour microenvironment (30, 31). Therefore, we propose that adipose tissue-derived EVs from obese mice may suppress splenocyte-mediated Panc02 cell death through the upregulation of IL-4. However, we were unable to determine whether IL-4 production originates in Panc02 cells or spleen cells. Consequently, further studies will be conducted to ascertain the expression of IL-4 in each cell type.

IL-2 and IL-12 are vital cytokines within the immune system, playing crucial roles in anti-cancer responses. They promote the proliferation and activation of T cells and NK cells, which are pivotal for an effective anti-tumour defence. These cytokines are potent, pro-inflammatory molecules that have long been under scrutiny as potential immunotherapy agents for cancer (32, 33). Consequently, we anticipated a reduction in these cytokines because of adipose tissue-derived EVs from obese mice. However, contrary to our expectations, EVs derived from the adipose tissue of obese mice actually led to an increase in the levels of IL-2 and IL-12 in the culture media from Panc02 cells co-cultured with splenocytes. Kochumon et al. and Suárez-Álvarez et al. demonstrated an upregulation of IL-2 and IL-12 in obese subjects, which is associated with inflammation and insulin resistance (34, 35). This is related to the previous studies that found that cancer immunotherapy utilising anti-CD40 and IL-2 in obese mice may lead to the induction of a cytokine storm, causing rapid lethality through the release of pro-inflammatory factors like TNF-α and IL-6 (36, 37). Therefore, we propose that adipose tissue-derived EVs from obese mice may play a role in driving the adverse effects of cancer immunotherapy.

This study is significant as it confirms, for the first time, the role of adipose tissue-derived EVs in understanding the complex relationship between obesity, cancer and immune responses. In the future, we will conduct research on the molecular mechanisms to determine the effects adipose tissue-derived EVs can have on cancer cells and immune cells, respectively.

## Conclusion

We suggest that adipose tissue-derived EVs from obese mice may play a role in suppressing splenocyte-mediated Panc02 cell death through the upregulation of IL-4, and in driving the adverse effects of cancer immunotherapy through the upregulation of IL-2 and IL-12p70. Our findings suggest a role for adipose tissue-derived EVs in mediating the interaction between obesity and cancer immunotherapy, offering a mechanistic insight into the intricate relationship between obesity, cancer and immune responses.
